# Author Correction: Climatic conditions are weak predictors of asylum migration

**DOI:** 10.1038/s41467-021-23945-9

**Published:** 2021-06-08

**Authors:** Sebastian Schutte, Jonas Vestby, Jørgen Carling, Halvard Buhaug

**Affiliations:** 1 Peace Research Institute Oslo, Oslo, Norway; 2 Department of Sociology and Political Science, Norwegian University of Science and Technology, Trondheim, Norway

**Keywords:** Developing world, Climate-change impacts, Society

Correction to: *Nature Communications* 10.1038/s41467-021-22255-4, published online 6 April 2021.

The original version of this Article contained several typographic errors. These have been corrected in both the PDF and HTML versions of the Article.

