# Supplementary material for: Climatic conditions are weak predictors of asylum migration
Source: Nat Commun. 2021 Apr 6;12:2067. doi: 10.1038/s41467-021-22255-4 (PMC8024373; doi:10.1038/s41467-021-22255-4)
Supplement: Supplementary file 1 — Supplementary Information [file 41467_2021_22255_MOESM1_ESM.pdf]

Supplementary Information for

Climatic conditions are weak predictors of asylum migration

Sebastian Schutte[1], Jonas Vestby[1], Jørgen Carling[1] & Halvard Buhaug[1,2*]

[1] Peace Research Institute Oslo, PO Box 9229 Grønland, 0134 Oslo, NORWAY
[2] Department of Sociology and Political Science, Norwegian University of Science and Technology, PO Box 8900 Torgarden, 7491 Trondheim, NORWAY
* Corresponding author: halvard@prio.org

Published in *Nature Communications*, 2021

Contents

1. Descriptive statistics 2
2. Additional documentation, main specification 4
  2.1. In-sample variable importance 4
  2.2. Interaction strength for temperature 5
3. Alternative Specifications and Sensitivity Tests 7
  3.1. No baseline indicators 7
  3.2. No time lag 9
  3.3. Two-year time lag 12
  3.4. Eight-year training period 15
  3.5. Twelve-year training period 18
  3.6. Prediction three years into the future 21
  3.7. Prediction eight years into the future 24
  3.8. Absolute asylum numbers 27
  3.9. Global prediction of asylum stocks 30
  3.10 In-sample regression analysis 33

1. Descriptive statistics

This document provides details on measurements and documentation of sensitivity tests to support the main results presented in the article. Supplementary Table 1 provides descriptive statistics; Supplementary Fig. 1 visualizes bivariate correlations between the variables.

Supplementary Table 1. Descriptive statistics

Variable	Mean	SD	Median	Min	Max
year	2008.6	5.77	2009	1999	2018
n_asylum_eu28	2,374.6	11,496.1	129	0	362,690
highest_neighbor_dem	0.71	0.21	0.76	0.00	0.95
area	11.91	2.03	12.12	5.71	16.61
wdi_pop	16.02	1.65	16.07	12.38	21.05
wdi_urban_pop	56.33	22.61	56.89	8.04	100.0
distance_to_eu	3,244.1	3,187.3	2,452.9	0.00	16,235.4
casualties_brd	1.03	2.36	0.00	0.00	10.61
annually_affected_20k	3.33	5.35	0.00	0.00	15.71
physical_integrity	0.67	0.27	0.74	0.02	0.99
free_movement	1.06	1.25	1.31	-4.21	2.95
homicide	1.71	0.88	1.57	0.35	4.73
wdi_gdppc_growth	0.02	0.05	0.02	-0.62	1.22
wdi_gdppc	9.09	1.23	9.20	6.30	11.73
kof_index	469.5	245.6	446.0	1.00	936.0
perc_post_secondary	15.68	12.23	13.96	0.09	86.94
wdi_imr	30.52	28.17	19.90	1.40	144.9
tmp_pop	18.69	7.10	20.54	-2.25	29.86
spei3_gs_pos	0.18	0.26	0.02	0.00	1.85
spei3_gs_neg	0.16	0.25	0.00	0.00	1.99
spei3_gs_pos_r3	0.18	0.16	0.14	0.00	0.99
spei3_gs_neg_r3	0.16	0.16	0.12	0.00	0.98

Number of countries: 175; number of country-years: 3,413.

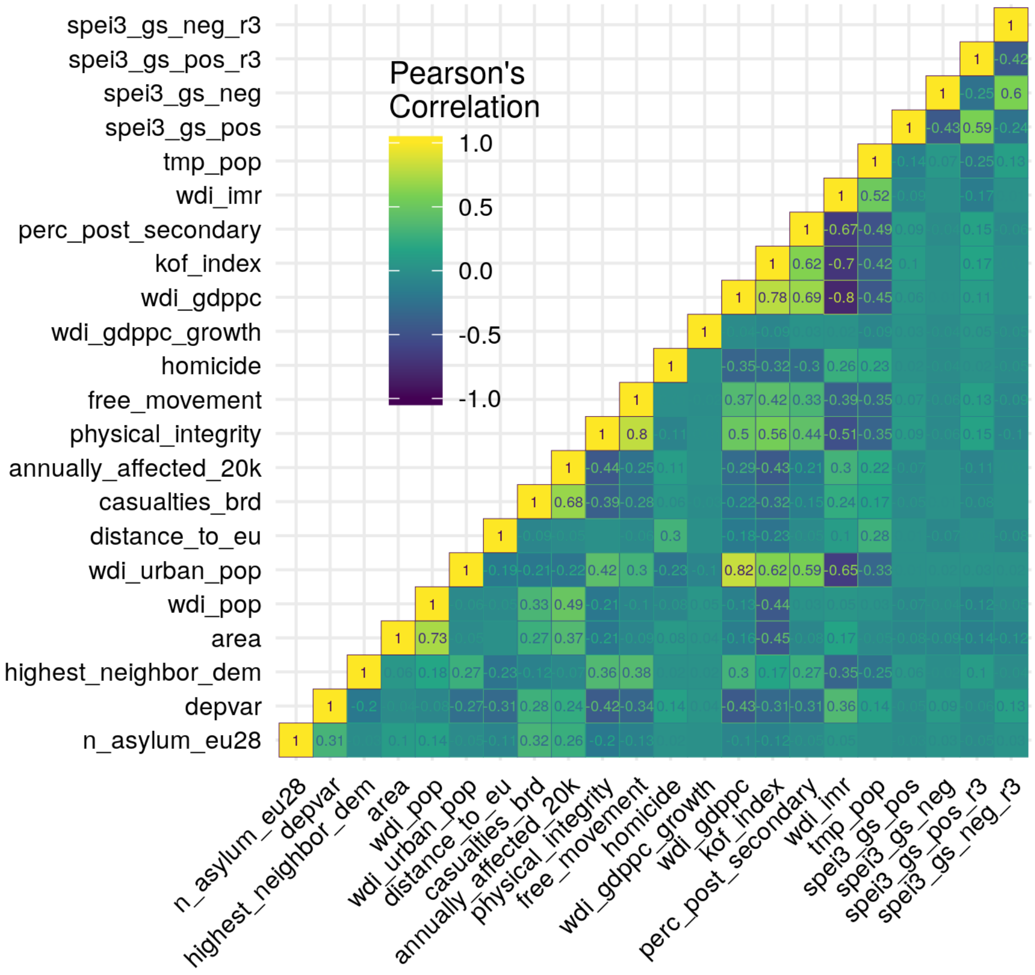

Supplementary Fig. 1. Correlation matrix

2. Additional documentation, main specification

2.1. In-sample variable importance

As a complement to the assessment of the predictive performance of component models (Fig. 3 in article) and the ALEs (Fig. 4 in article), we here calculate, for each indicator, how much the complete model's in-sample mean square error (MSE) would increase if we permute the input data for the given indicator. We repeat this test for all training samples for all alternative imputation values in order to obtain a distribution of importance estimates (Supplementary Fig. 2). In line with the ALE, we find that several static or inert indicators score well in this test, since they capture underlying structural conditions (e.g., geography, demography) that shape the potential for exporting asylum seekers to Europe. Temperature also ranks quite highly by this metric, compared to the modest performance of the climate model and the temperature ALE. The time-varying drought measures, which revealed very weak ALEs, also contribute little to improving the model's recall. Variable importance scores like these should be interpreted carefully as correlated variables can steal or lend importance to each other (e.g., temperature may capture inert structural conditions that shape latent production of asylum seekers, such that out-of-sample predictive power is lower than variable importance might suggest).

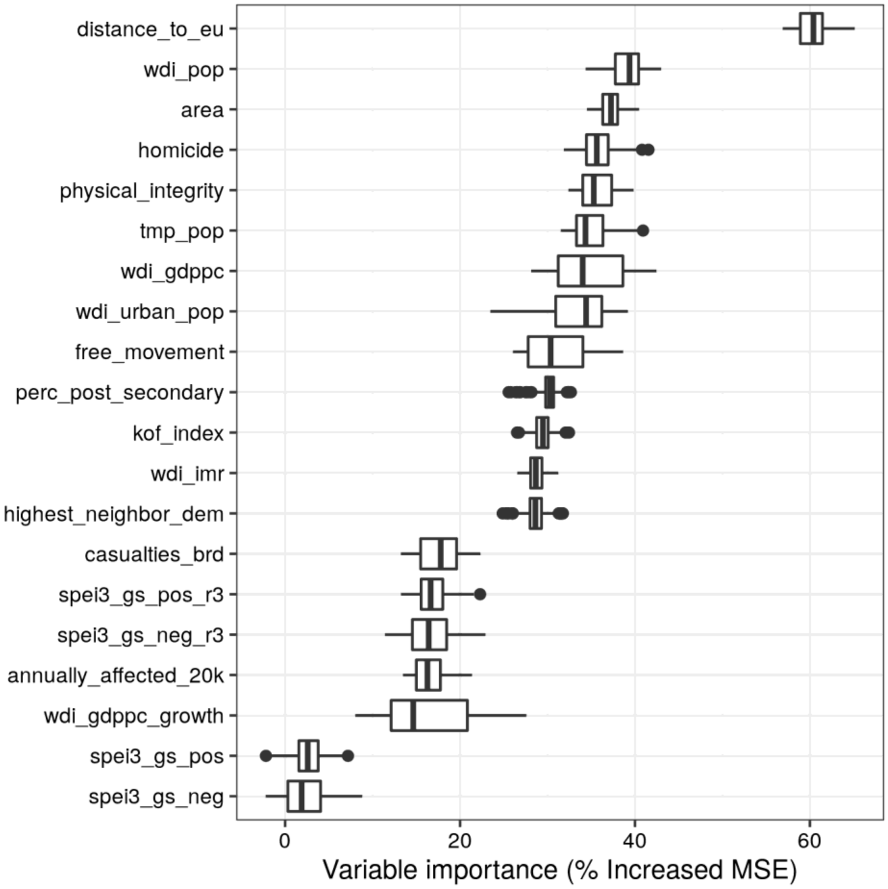

Supplementary Fig. 2. Variable importance plot; main specification. Boxes range from first to third quartile with interior line marking the median, whiskers denote 1.5 interquartile range, and dots represent outliers. MSE is mean square error. N = 3,413 country-year observations, examined over 160 simulations.

2.2. Interaction strength for temperature

To shed further light on the behavior of temperature, the most relevant climate indicator in our analysis, we identify the most important factors that condition the predictive effect of temperature. Unlike assumptions that climate is a relevant driver of asylum migration through its influence conflict risk and dynamics[1,2], we find that temperature interacts most often with core economic and political conditions (Supplementary Fig. 3). These plots do not reveal the shape of those interactions, however. We defer a deeper probe into conditional determinants of climate-migration links to future research.

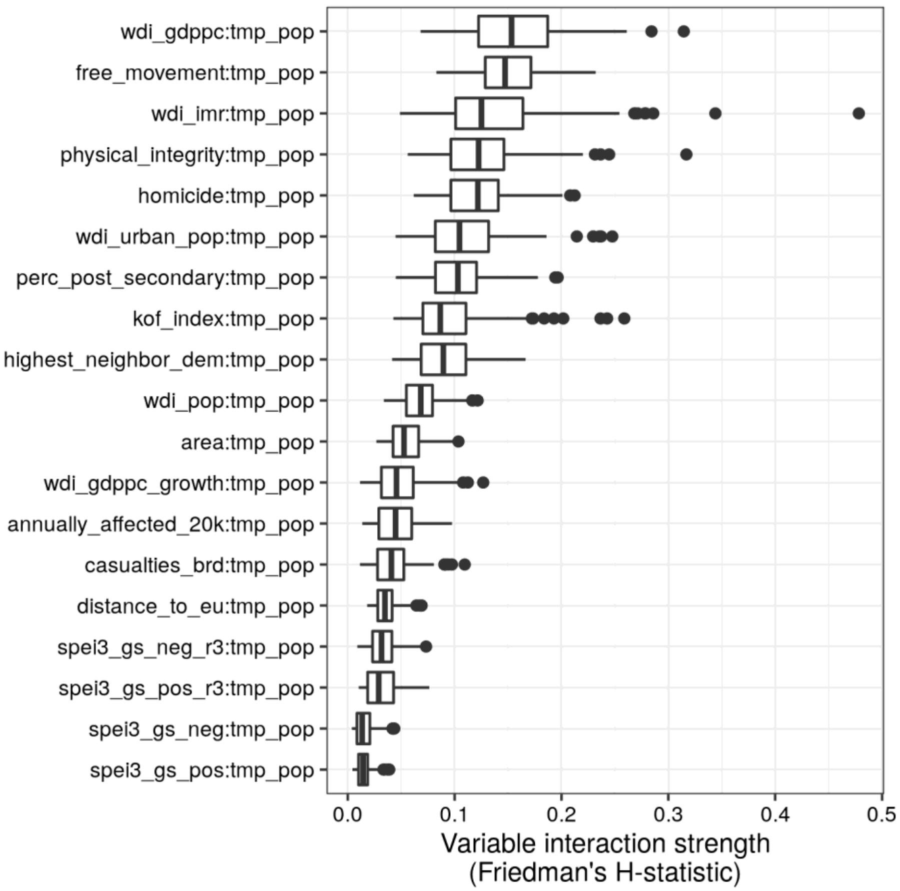

Supplementary Fig. 3. Main interactions for temperature. Boxes range from first to third quartile with interior line marking the median, whiskers denote 1.5 interquartile range, and dots represent outliers. N = 3,413 country-year observations, examined over 160 simulations.

3. Alternative Specifications and Sensitivity Tests

Here, we present results from a set of sensitivity tests where we iteratively alter central aspects of the preferred model specification. We also document results from in-sample regression models.

3.1. No baseline indicators

We begin by inspecting the predictive performance of the thematic components without the common baseline indicators. The baseline indicators are largely static and only shape structural conditions that can favor, or impede, fleeing to Europe, and do not contribute to explaining the dramatic surge in new asylum migrants in recent years (although their inclusion very likely conditions the behavior of other predictors). When these are dropped, temperature becomes the most influential variable in terms of reducing model's in-sample mean square errors (Supplementary Fig. 5), because it now captures more of the static geographical determinants of migration opportunities. Yet, the climate component as a whole performs poorly in predicting asylum migration on new data and barely manages to separate between low- and high-volume cases (Supplementary Fig. 4). The economy model behaves more in line with the violence model, but the latter demonstrates its predictive superiority also when basic structural controls are removed.

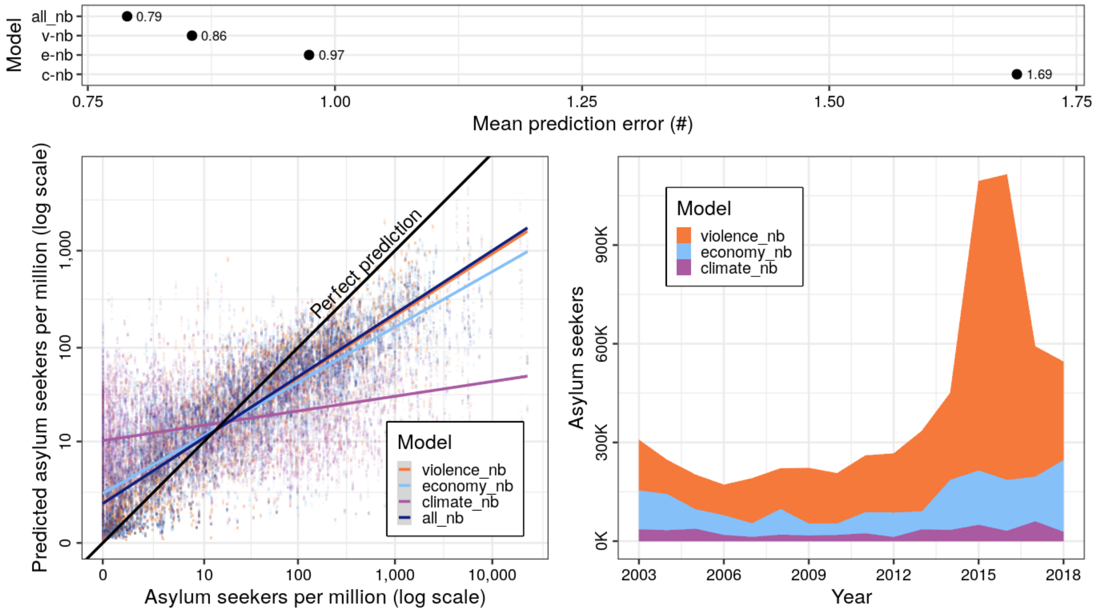

Supplementary Fig. 4. Out-of-sample prediction performance; no baseline. Results are generated from leave-future-out cross-validation, trained on alternative four-year subsets of empirical data for the period 1999–2017 and tested against observed outcomes for the subsequent year, 2003–2018. N = 3,413 country-year observations, examined over 160 simulations.

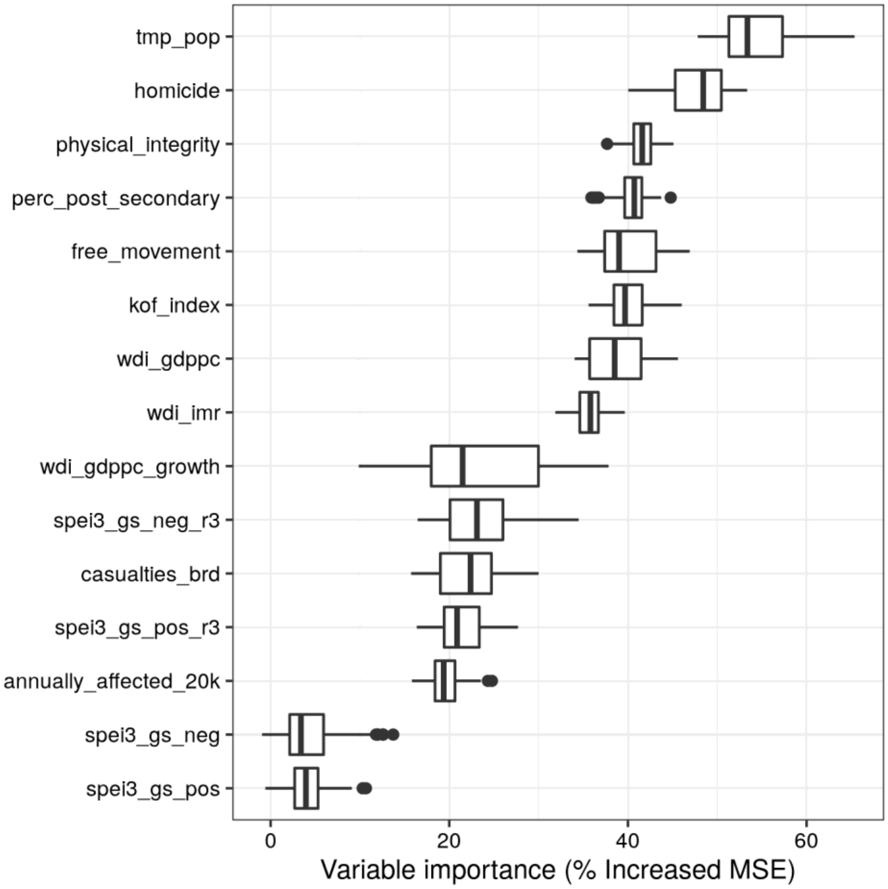

Supplementary Fig. 5. Variable importance plot; no baseline. Boxes range from first to third quartile with interior line marking the median, whiskers denote 1.5 interquartile range, and dots represent outliers. MSE is mean square error. N = 3,413 country-year observations, examined over 160 simulations.

3.2. No time lag

In the main specification, all component indicators are lagged one year to allow a slight delay in the response and to ensure that the predictors measure conditions prior to the asylum statistics. Here, we consider the predictive performance of the models using contemporaneous indicators. While this specification is at odds with reports about durable migrant transit routes and, further, runs the risk of measuring the predictors partly prior to when the response is observed, it is preferred in some earlier research[2]. As shown in Supplementary Figs. 6–8, the results remain substantively unchanged; the violence model predicts best on average and also performs better than the other models in capturing the large inflow on migrants to Europe since 2014 whereas the climate component is associated with the highest average prediction error.

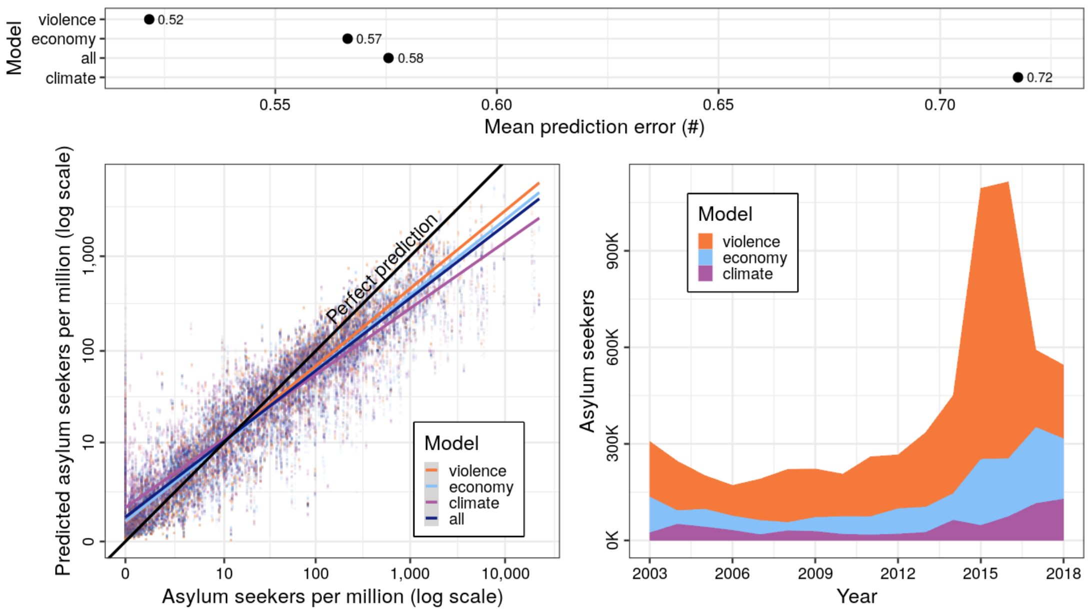

Supplementary Fig. 6. Out-of-sample prediction performance; no time lag. Results are generated from leave-future-out cross-validation, trained on alternative four-year subsets of empirical data for the period 1999–2017 and tested against observed outcomes for the subsequent year, 2003–2018. N = 3,413 country-year observations, examined over 160 simulations.

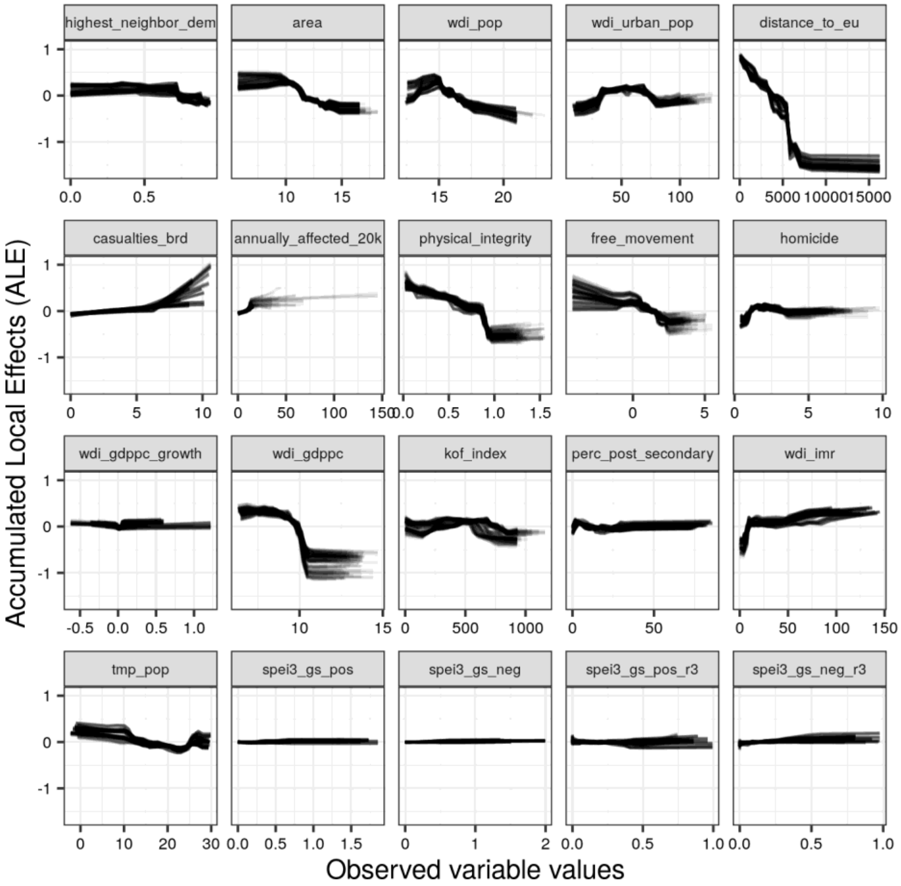

Supplementary Fig. 7. Accumulated Local Effects (ALE); no time lag. ALEs give the marginal difference in prediction with an incremental change in the predictor. Y-axis values represent change in log asylum applications per capita. The results are generated from leave-future-out cross-validation, trained on alternative four-year subsets of empirical data for the period 1999–2017 and tested against observed outcomes for the same year, 2003–2018. N = 3,413 country-year observations, examined over 160 simulations.

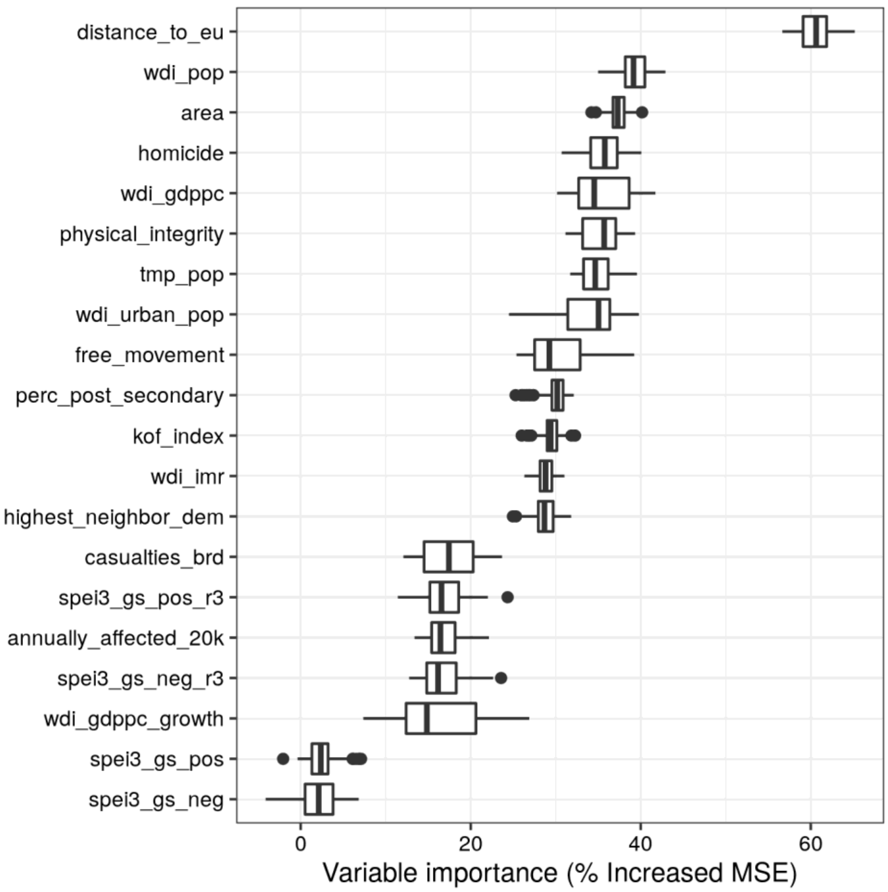

Supplementary Fig. 8. Variable importance plot; no time lag. Boxes range from first to third quartile with interior line marking the median, whiskers denote 1.5 interquartile range, and dots represent outliers. MSE is mean square error. N = 3,413 country-year observations, examined over 160 simulations.

3.3. Two-year time lag

In the main specification, all component indicators are lagged one year to allow a slight delay in the response and to ensure that the predictors measure conditions prior to the asylum statistics. However, it often takes considerable time from a decision to flee is made until the migrant arrives in Europe as transit routes often are circuitous and protracted[3]. One survey found that 34% of arrivals in Italy and Malta had left their country of origin more than 18 months earlier[4]. Besides, many migrants to Europe avoid seeking asylum in the country of first arrival but instead try to move on to their preferred destination (e.g., Germany or Sweden) before filing their application[5]. Supplementary Figs. 9–11 reveal that the main results are robust to specifying two-year time lags to the predictors.

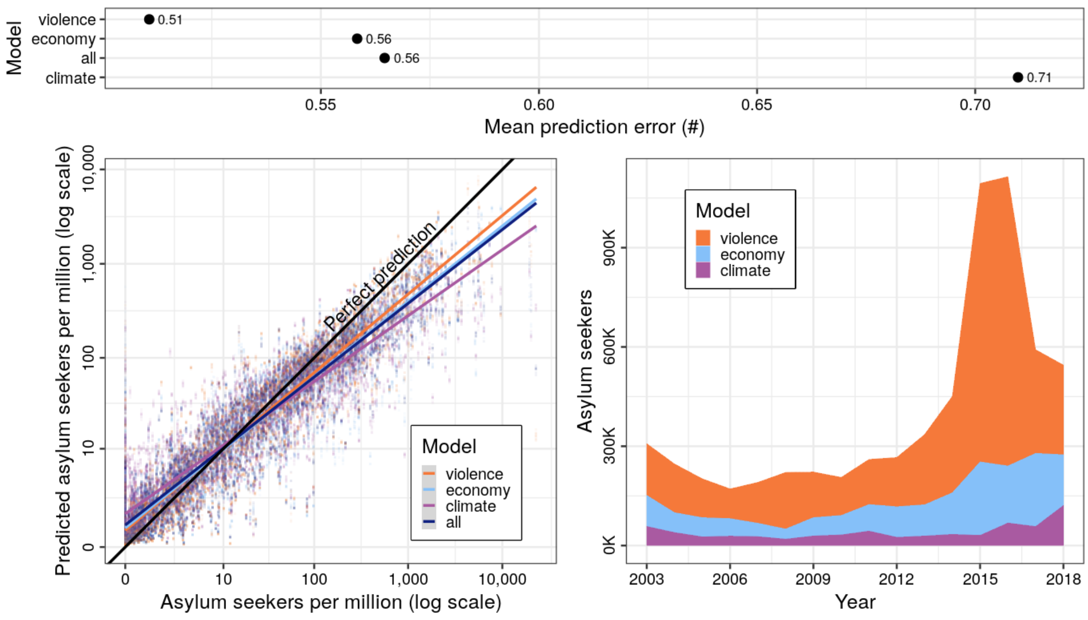

Supplementary Fig. 9. Out-of-sample prediction performance; two-year time lag. Results are generated from leave-future-out cross-validation, trained on alternative four-year subsets of empirical data for the period 1999–2017 and tested against observed outcomes for the subsequent year, 2003–2018. N = 3,413 country-year observations, examined over 160 simulations.

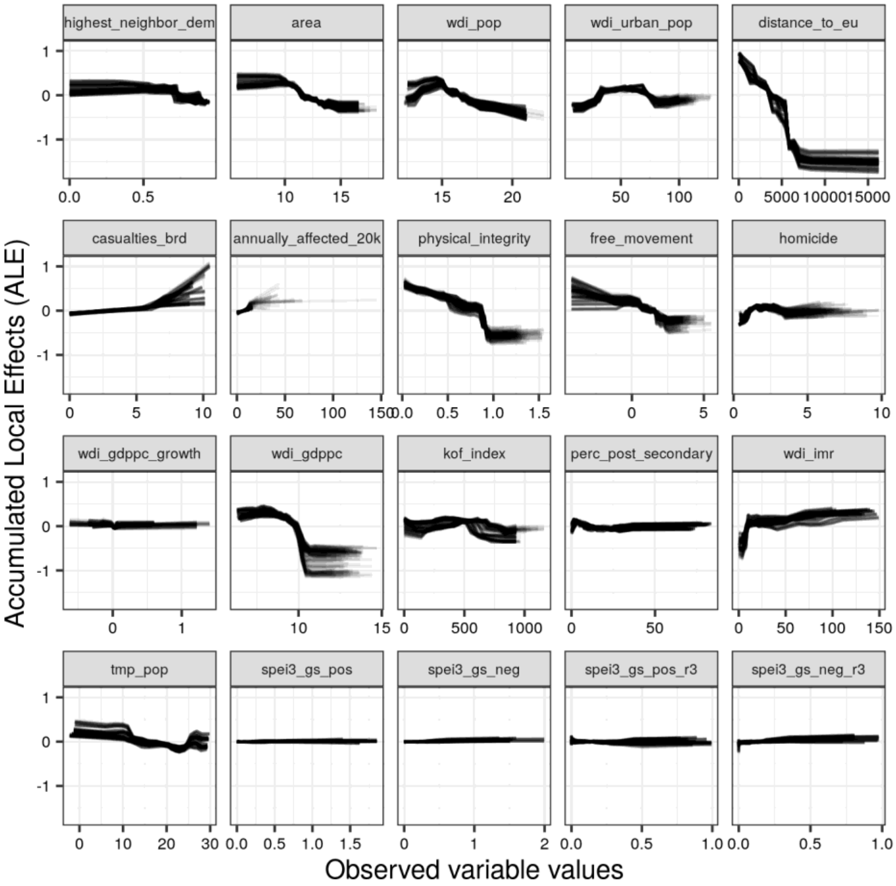

Supplementary Fig. 10. Accumulated Local Effects (ALE); two-year time lag. ALEs give the marginal difference in prediction with an incremental change in the predictor. Y-axis values represent change in log asylum applications per capita. The results are generated from leave-future-out cross-validation, trained on alternative four-year subsets of empirical data for the period 1999–2017 and tested against observed outcomes for the subsequent year, 2003–2018. N = 3,413 country-year observations, examined over 160 simulations.

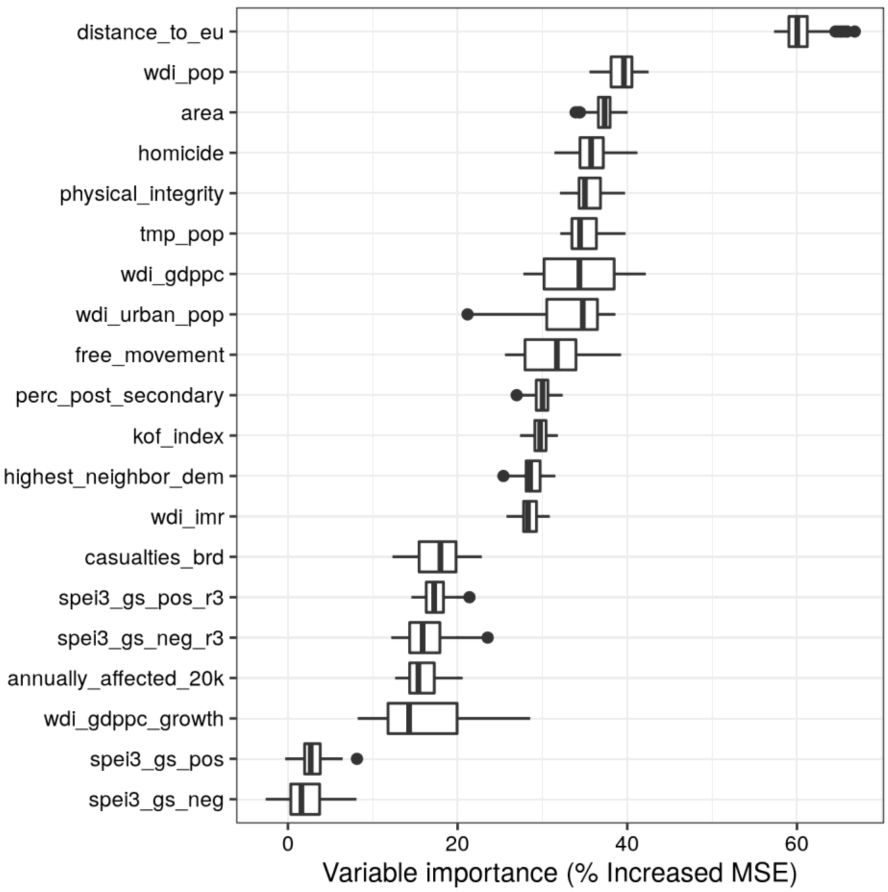

Supplementary Fig. 11. Variable importance plot; two-year time lag. Boxes range from first to third quartile with interior line marking the median, whiskers denote 1.5 interquartile range, and dots represent outliers. MSE is mean square error. N = 3,413 country-year observations, examined over 160 simulations.

3.4. Eight-year training period

In the main specification, models were trained on sliding four-year periods of empirical data, and then evaluated against observed outcomes for the subsequent year. Here, we extend the training periods to eight years (1999–2006, 2000–07, 2001–08, …, 2010–17), predicting on the subsequent year, 2007–18, to allow model parameters to be informed by more data points. Even so, the new predictions (Supplementary Figs. 12–14) are similar to, but not more accurate on average than, the main models that only use four years of data for each prediction.

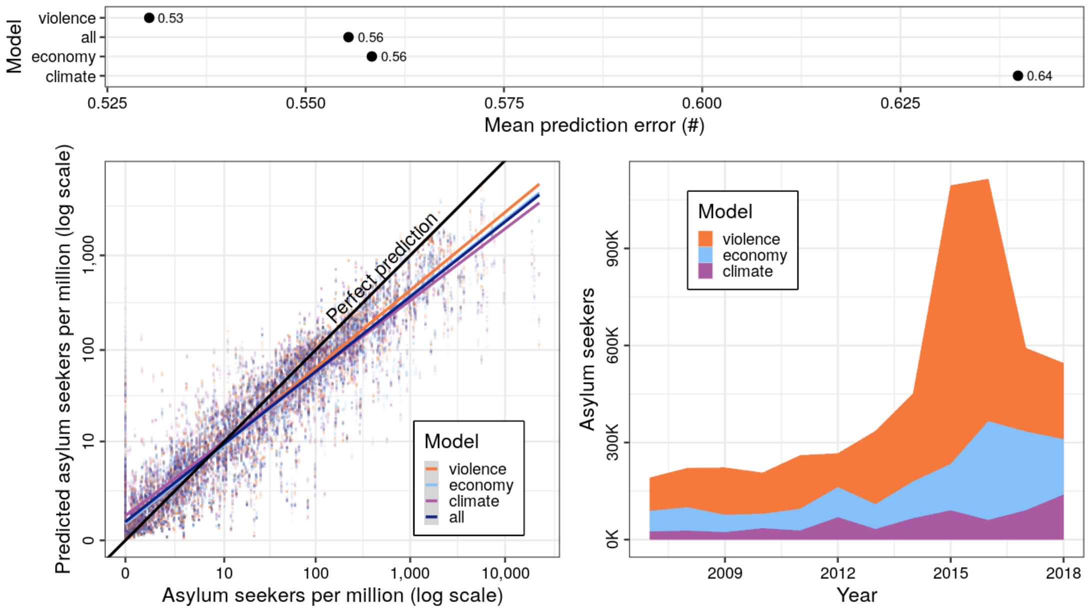

Supplementary Fig. 12. Out-of-sample prediction performance; eight-year training period. Results are generated from leave-future-out cross-validation, trained on alternative eight-year subsets of empirical data for the period 1999–2017 and tested against observed outcomes for the subsequent year, 2007–2018. N = 3,413 country-year observations, examined over 120 simulations.

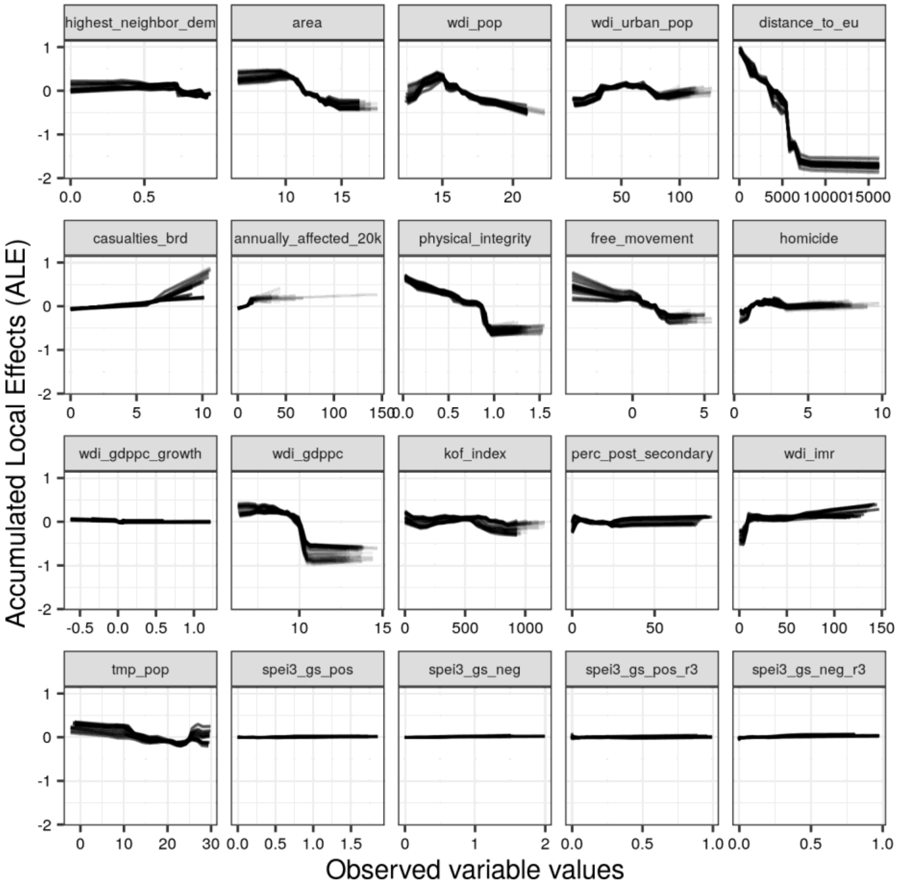

Supplementary Fig. 13. Accumulated Local Effects (ALE); eight-year training period. ALEs give the marginal difference in prediction with an incremental change in the predictor. Y-axis values represent change in log asylum applications per capita. The results are generated from leave-future-out cross-validation, trained on alternative eight-year subsets of empirical data for the period 1999–2017 and tested against observed outcomes for the subsequent year, 2007–2018. N = 3,413 country-year observations, examined over 120 simulations.

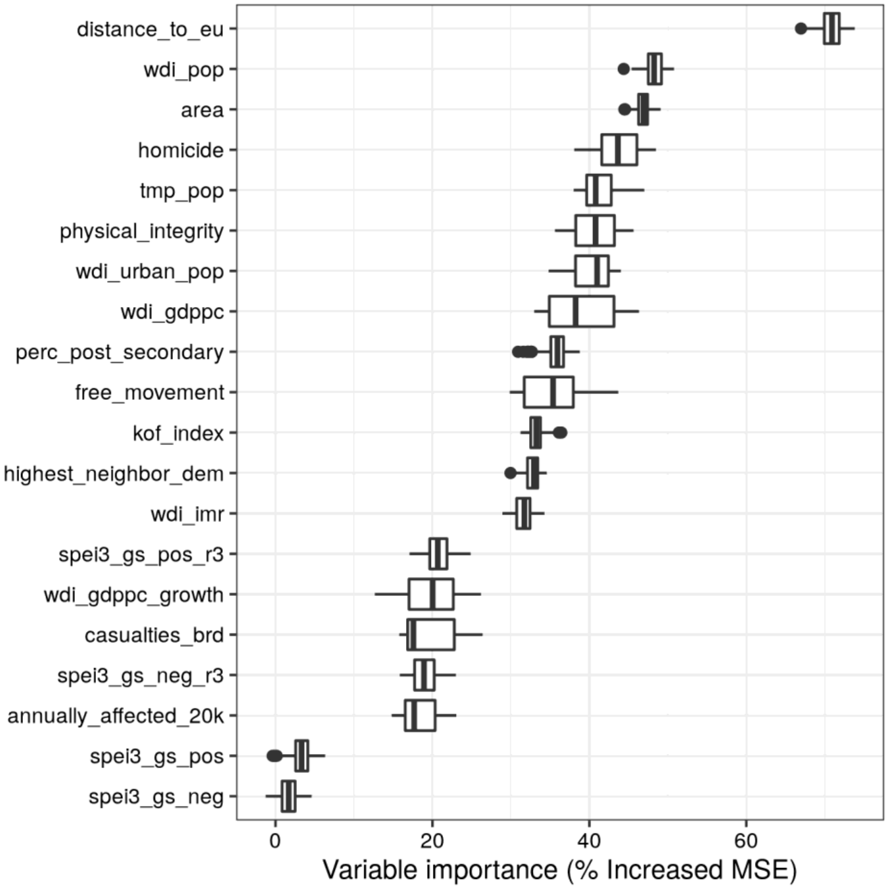

Supplementary Fig. 14. Variable importance plot; eight-year training period. Boxes range from first to third quartile with interior line marking the median, whiskers denote 1.5 interquartile range, and dots represent outliers. MSE is mean square error. N = 3,413 country-year observations, examined over 120 simulations.

3.5. Twelve-year training period

In the main specification, models were trained on sliding four-year periods of empirical data, and then evaluated against observed outcomes for the subsequent year. Here, we extend the training periods to 12 years (1999–2010, 2000–11, 2001–12, …, 2006–17), predicting on the subsequent year, 2011–18, to allow model parameters to be informed by even more data points. As shown in Supplementary Figs. 15–17, the violence model now performs slightly worse than in the main specification (Fig. 3 in the article), although the ranking of the component models remains unaffected.

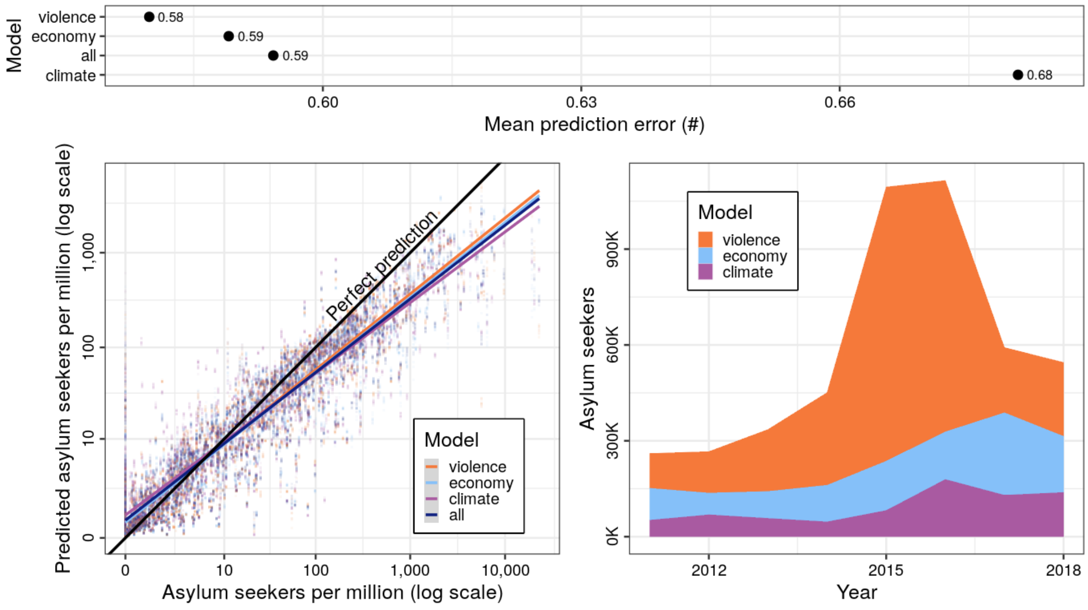

Supplementary Fig. 15. Out-of-sample prediction performance; twelve-year training period. Results are generated from leave-future-out cross-validation, trained on alternative twelve-year subsets of empirical data for the period 1999–2017 and tested against observed outcomes for the subsequent year, 2011–2018. N = 3,413 country-year observations, examined over 80 simulations.

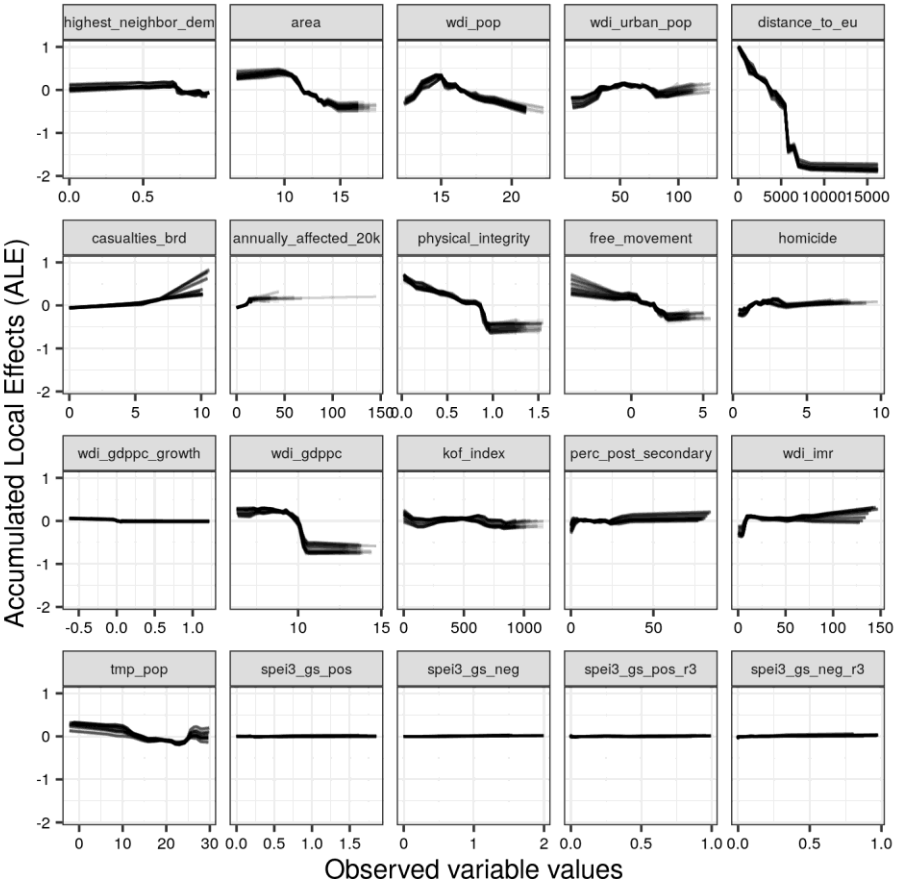

Supplementary Fig. 16. Accumulated Local Effects (ALE); twelve-year training period. ALEs give the marginal difference in prediction with an incremental change in the predictor. Y-axis values represent change in log asylum applications per capita. The results are generated from leave-future-out cross-validation, trained on alternative twelve-year subsets of empirical data for the period 1999–2017 and tested against observed outcomes for the subsequent year, 2011–2018. N = 3,413 country-year observations, examined over 80 simulations.

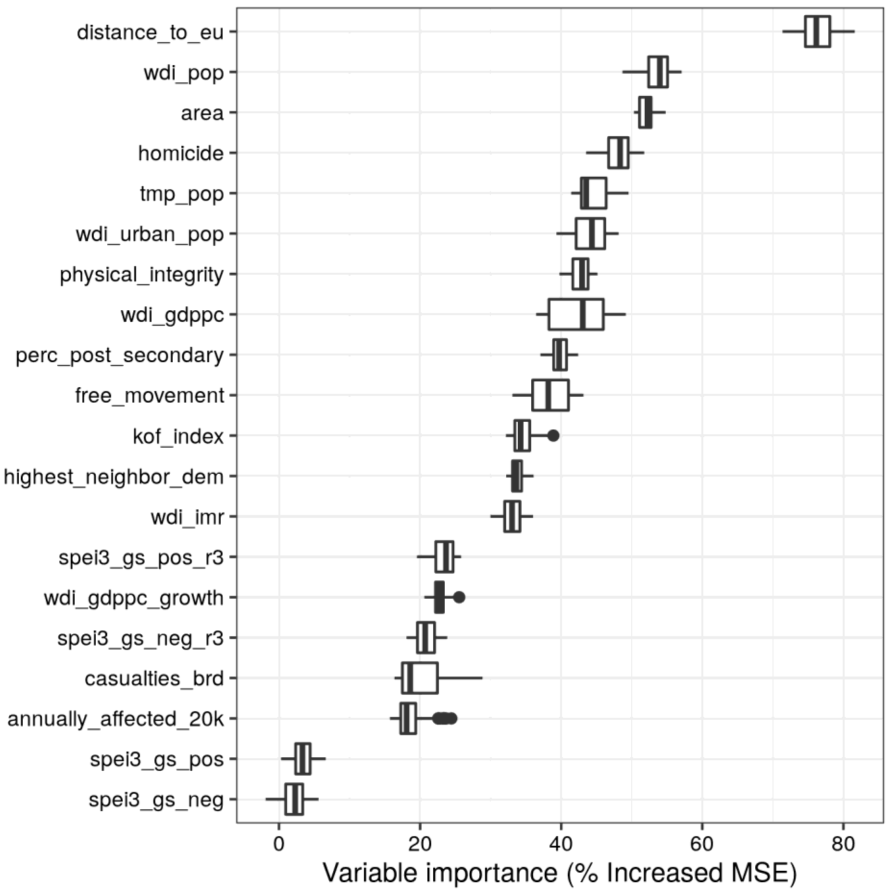

Supplementary Fig. 17. Variable importance plot; twelve-year training period. Boxes range from first to third quartile with interior line marking the median, whiskers denote 1.5 interquartile range, and dots represent outliers. MSE is mean square error. N = 3,413 country-year observations, examined over 80 simulations.

3.6. Prediction three years into the future

In the main specification, model performance was evaluated based on a comparison between predictions and observed outcomes for the year immediately following the training period. Here, we introduce a temporal gap between the training and test samples by instead predicting asylum migration three years into the future. This is a more demanding exercise, and model performance, represented by mean prediction error, deteriorates. Yet, the violence model again demonstrates that it is better able to predict new asylum migration flows than the economy and climate models (Supplementary Figs. 18–20).

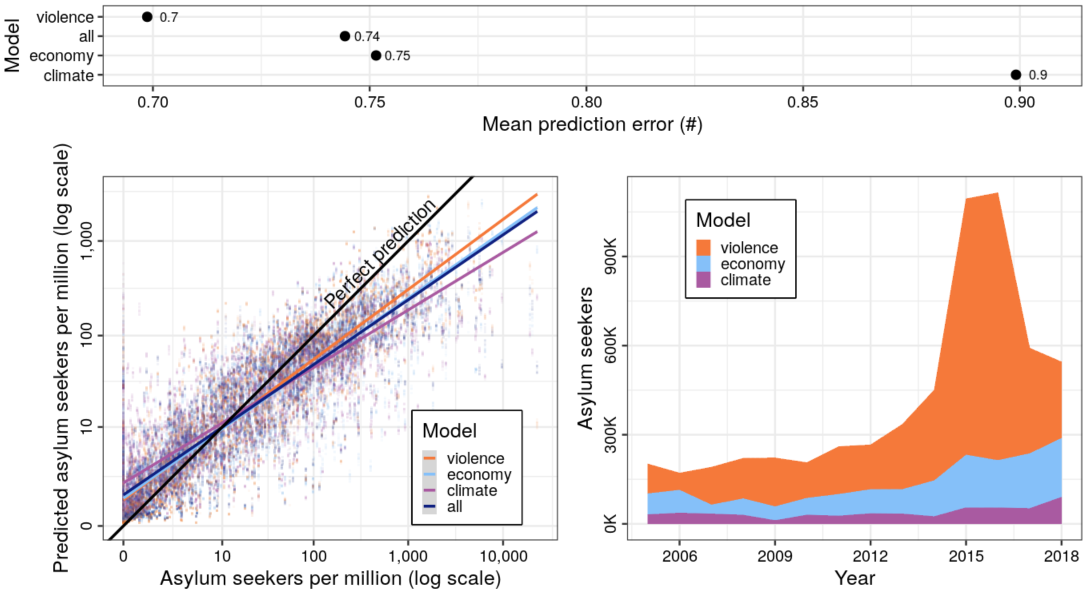

Supplementary Fig. 18. Out-of-sample prediction performance; prediction three years into the future. Results are generated from leave-future-out cross-validation, trained on alternative four-year subsets of empirical data for the period 1999–2017 and tested against observed outcomes three years later, 2005–2018. N = 3,413 country-year observations, examined over 140 simulations.

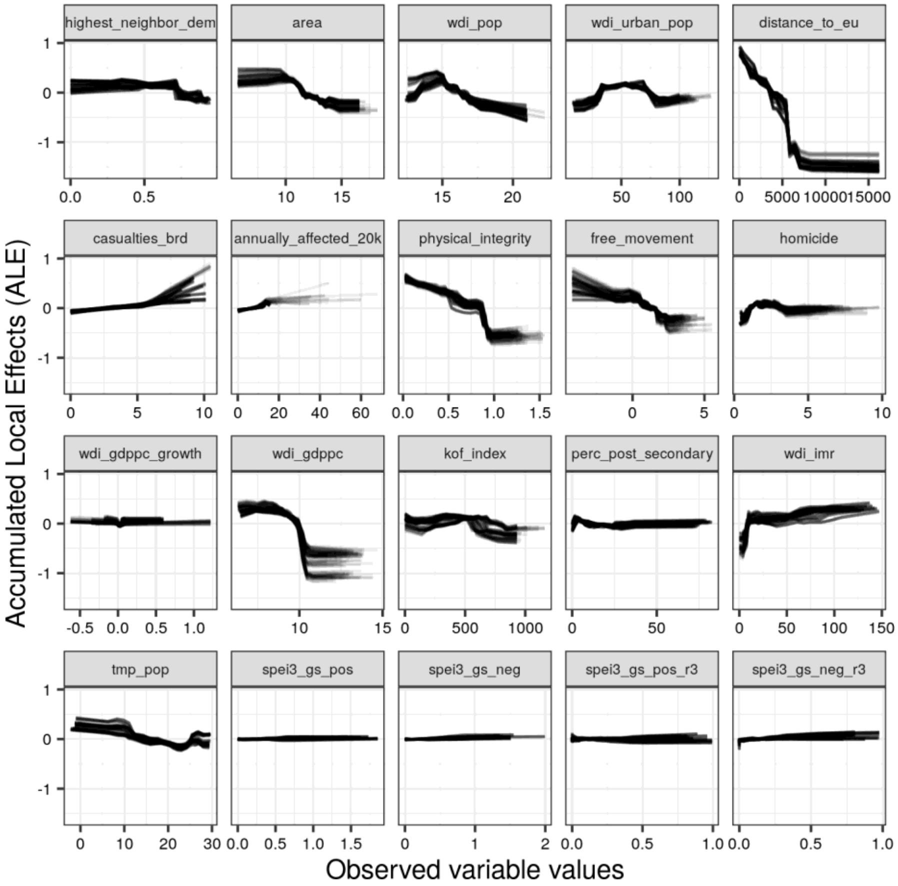

Supplementary Fig. 19. Accumulated Local Effects (ALE); prediction three years into the future. ALEs give the marginal difference in prediction with an incremental change in the predictor. Y-axis values represent change in log asylum applications per capita. The results are generated from leave-future-out cross-validation, trained on alternative four-year subsets of empirical data for the period 1999–2015 and tested against observed outcomes three years later, 2005–2018. N = 3,413 country-year observations, examined over 140 simulations.

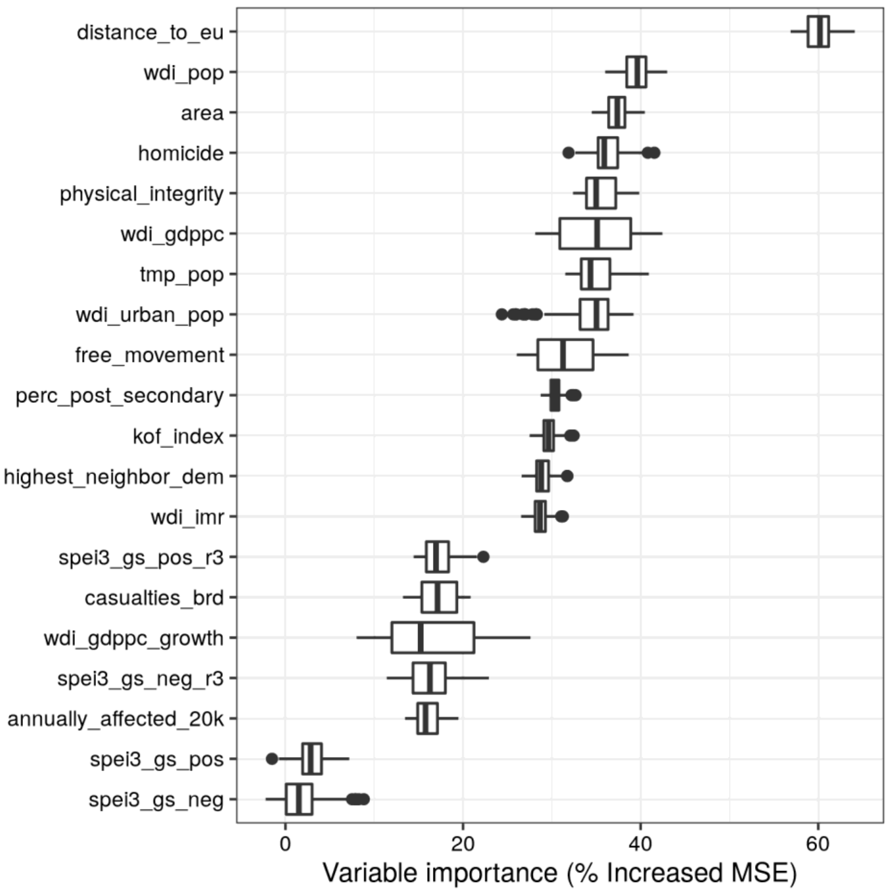

Supplementary Fig. 20. Variable importance plot; prediction three years into the future. Boxes range from first to third quartile with interior line marking the median, whiskers denote 1.5 interquartile range, and dots represent outliers. MSE is mean square error. N = 3,413 country-year observations, examined over 140 simulations.

3.7. Prediction eight years into the future

In the main specification, model performance was evaluated based on a comparison between predictions and observed outcomes for the year immediately following the training period. Here, we introduce a large temporal gap between the training and test samples by instead predicting asylum migration eight years into the future. This is the most demanding change in model specification among the sensitivity tests, but again the results (Supplementary Figs. 21–23) are in line with those presented in the article. Notably, the violence component is best able to capture the uptick in asylum migration since 2014 even when the model training period ends in 2010.

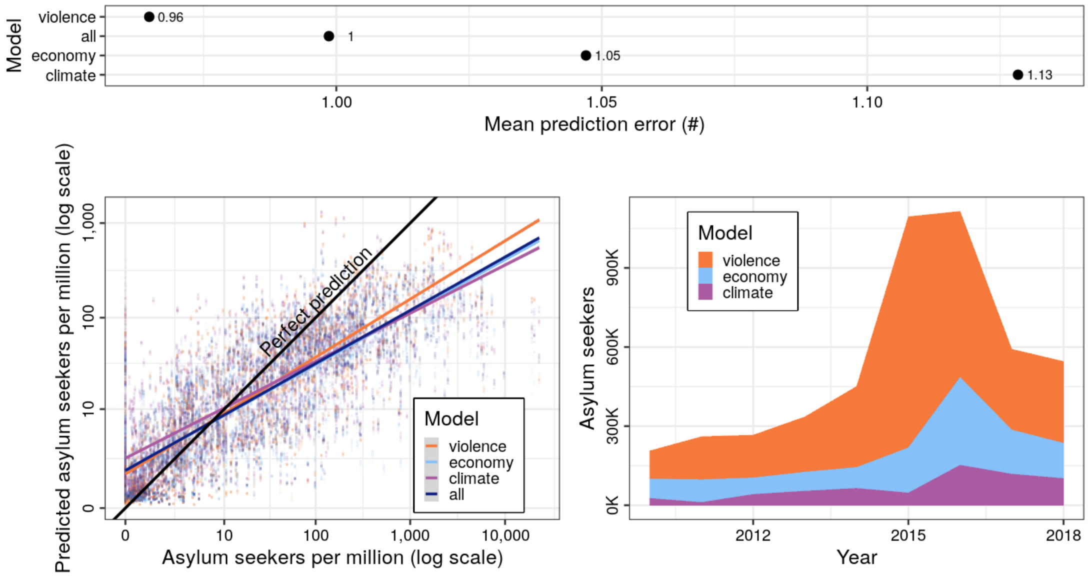

Supplementary Fig. 21. Out-of-sample prediction performance; prediction eight years into the future. Results are generated from leave-future-out cross-validation, trained on alternative four-year subsets of empirical data for the period 1999–2010 and tested against observed outcomes eight years later, 2010–2018. N = 3,413 country-year observations, examined over 90 simulations.

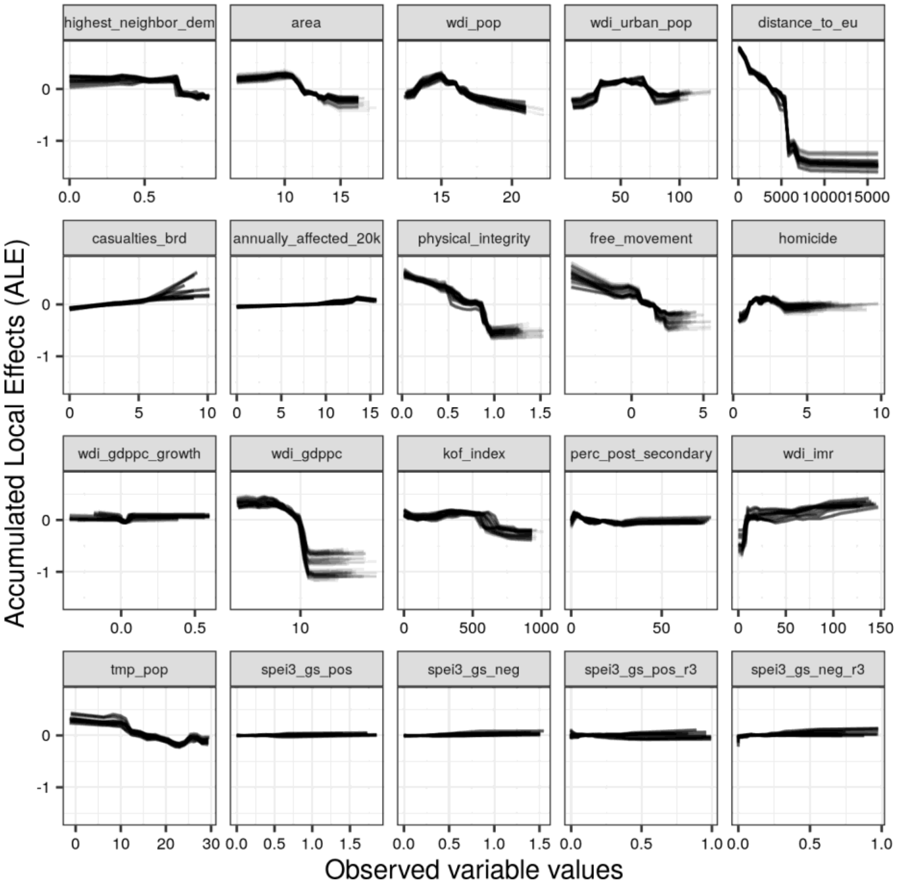

Supplementary Fig. 22. Accumulated Local Effects (ALE); prediction eight years into the future. ALEs give the marginal difference in prediction with an incremental change in the predictor. Y-axis values represent change in log asylum applications per capita. The results are generated from leave-future-out cross-validation, trained on alternative four-year subsets of empirical data for the period 1999–2010 and tested against observed outcomes eight years later, 2010–2018. N = 3,413 country-year observations, examined over 90 simulations.

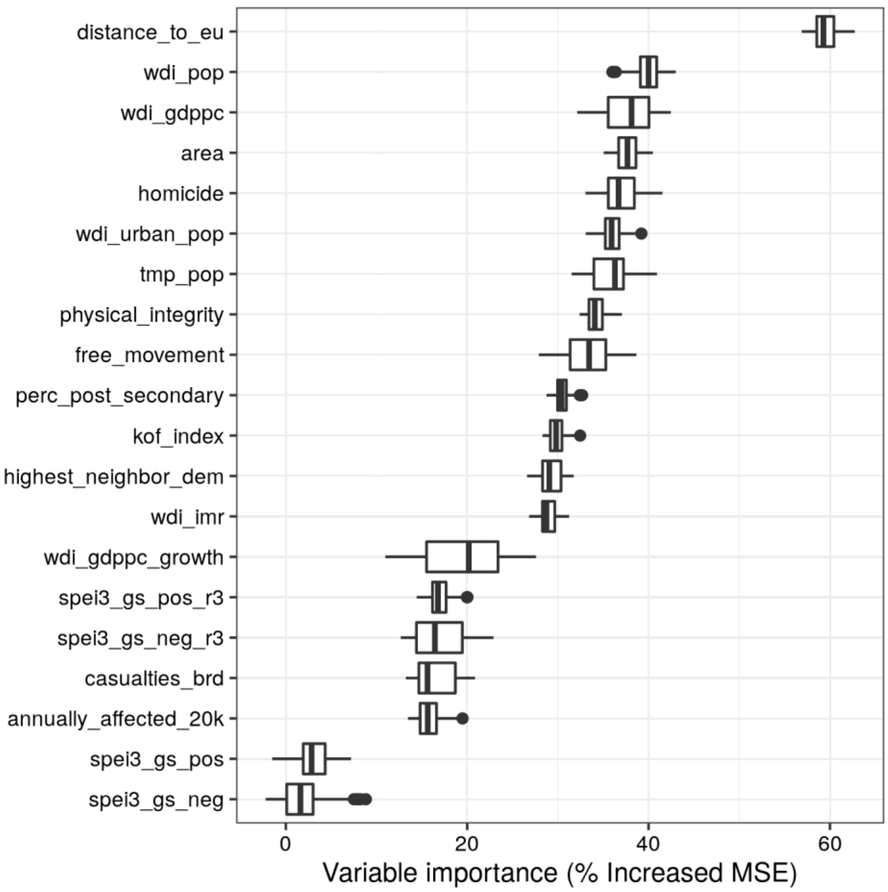

Supplementary Fig. 23. Variable importance plot; prediction eight years into the future. Boxes range from first to third quartile with interior line marking the median, whiskers denote 1.5 interquartile range, and dots represent outliers. MSE is mean square error. N = 3,413 country-year observations, examined over 90 simulations.

3.8. Absolute asylum numbers

In the main specification, the outcome variable gives the log-transformed rate of asylum applicants per capita. Here, we consider the models' ability to predict non-transformed raw asylum numbers, which is particularly relevant if we are most interested in finding the component model (and indicators) that best predict the largest flows of asylum migrants. In line with the main result, we find that the violence model is superior to its competitors (Supplementary Fig. 24). The economy model struggles more, and the linear prediction now indicates that it is marginally worse than the climate model on average. A similar impression is left by the indicator performance plots, where the two conflict severity variables (battle-deaths and number affected) now are highly influential (Supplementary Figs. 25–26).

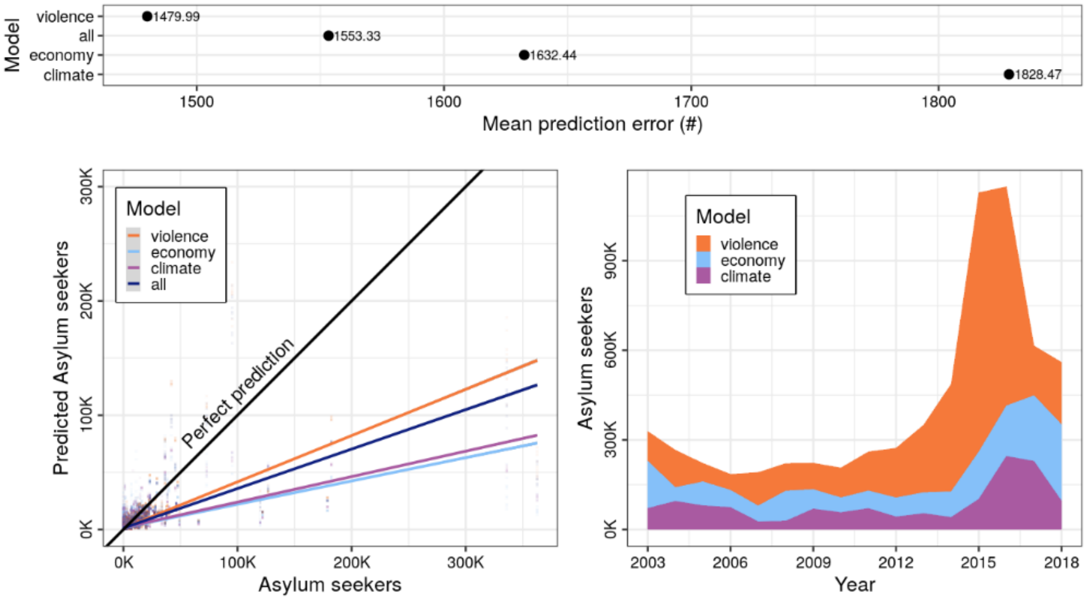

Supplementary Fig. 24. Out-of-sample prediction performance; absolute asylum numbers. Results are generated from leave-future-out cross-validation, trained on alternative four-year subsets of empirical data for the period 1999–2017 and tested against observed outcomes for the subsequent year, 2003–2018. N = 3,413 country-year observations, examined over 160 simulations.

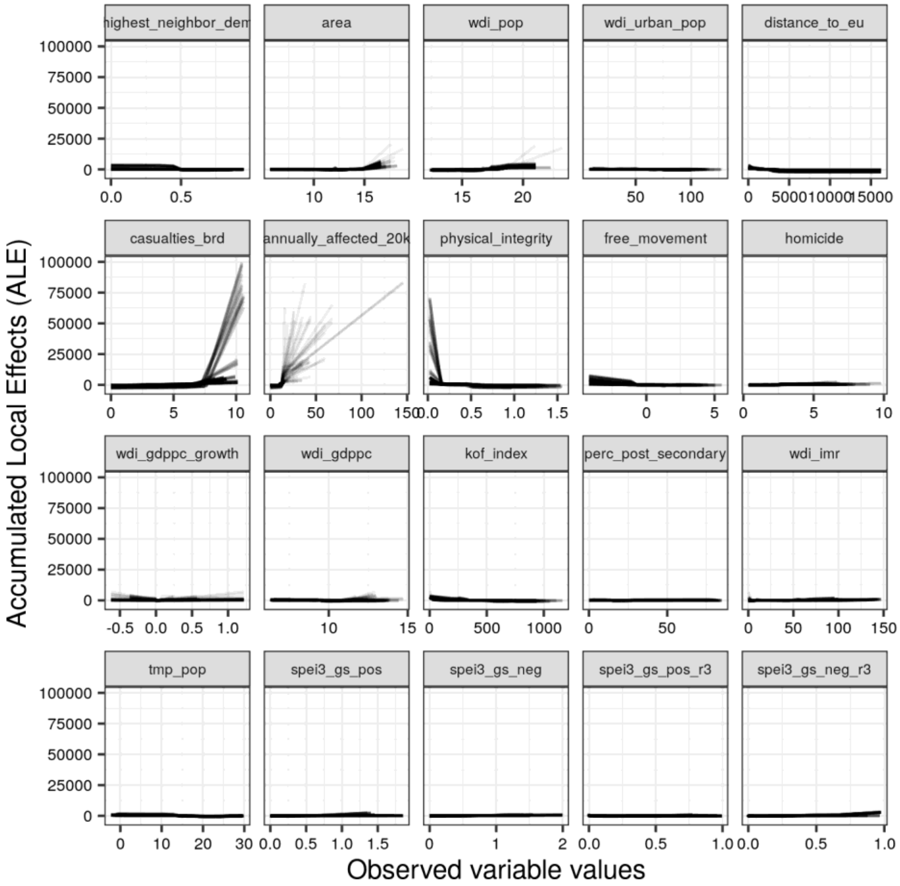

Supplementary Fig. 25. Accumulated Local Effects (ALE); absolute asylum numbers. ALEs give the marginal difference in prediction with an incremental change in the predictor. Y-axis values represent change in log asylum applications per capita. The results are generated from leave-future-out cross-validation, trained on alternative four-year subsets of empirical data for the period 1999–2017 and tested against observed outcomes for the subsequent year, 2003–2018. N = 3,413 country-year observations, examined over 160 simulations.

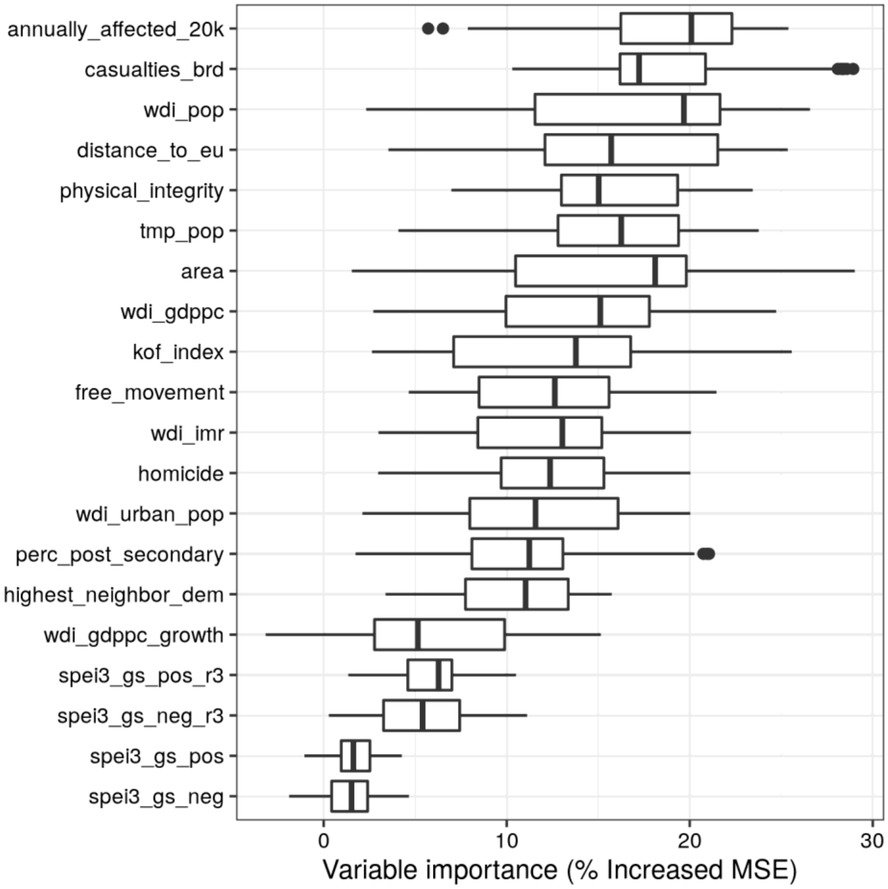

Supplementary Fig. 26. Variable importance plot; absolute asylum numbers. Boxes range from first to third quartile with interior line marking the median, whiskers denote 1.5 interquartile range, and dots represent outliers. MSE is mean square error. N = 3,413 country-year observations, examined over 160 simulations.

3.9. Global prediction of asylum stocks

In the main specification, the dependent variable measures the annual flow of new asylum seekers to the EU from each country of origin. The EU statistics are particularly attractive as they contain information on date of first arrival, which permits studying dynamic migration flows. Besides, EU member states have relatively streamlined asylum procedures. Elsewhere, the capacity, rules, and routines for handling transnational migrants requesting asylum vary widely[6]. Yet, patterns of asylum migration to Europe may not generalize to the world. Here, we consider global prediction models where the outcome is the annual stock of asylum seekers from each country or origin.

Globally, the number of asylum seekers continued to increase after 2016 (until the COVID-19 pandemic). This is partly a result of accumulating numbers of migrants awaiting decision on their asylum applications and partly a reflection of the recent contraction of the Venezuelan economy – the largest collapse outside of war in modern history according to the *New York Times* – which has forced people to cross borders in large numbers in seek of food, medicine, and shelter[7]. These global trends explain why the economy model is able to predict a larger share of the global volume of asylum seekers since 2016. Even so, the violence model overall is better able to predict future levels of asylum applications than the other component models (Supplementary Figs. 27–29).

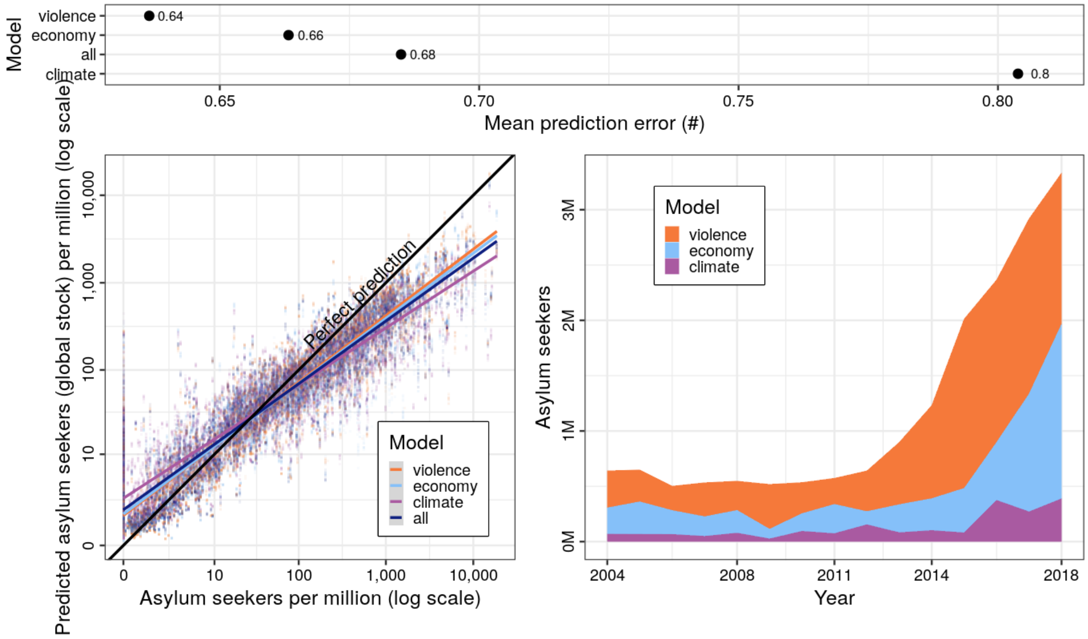

Supplementary Fig. 27. Out-of-sample prediction performance; global asylum stocks. Results are generated from leave-future-out cross-validation, trained on alternative four-year subsets of empirical data for the period 1999–2017 and tested against observed outcomes for the subsequent year, 2003–2018. N = 3,413 country-year observations, examined over 160 simulations.

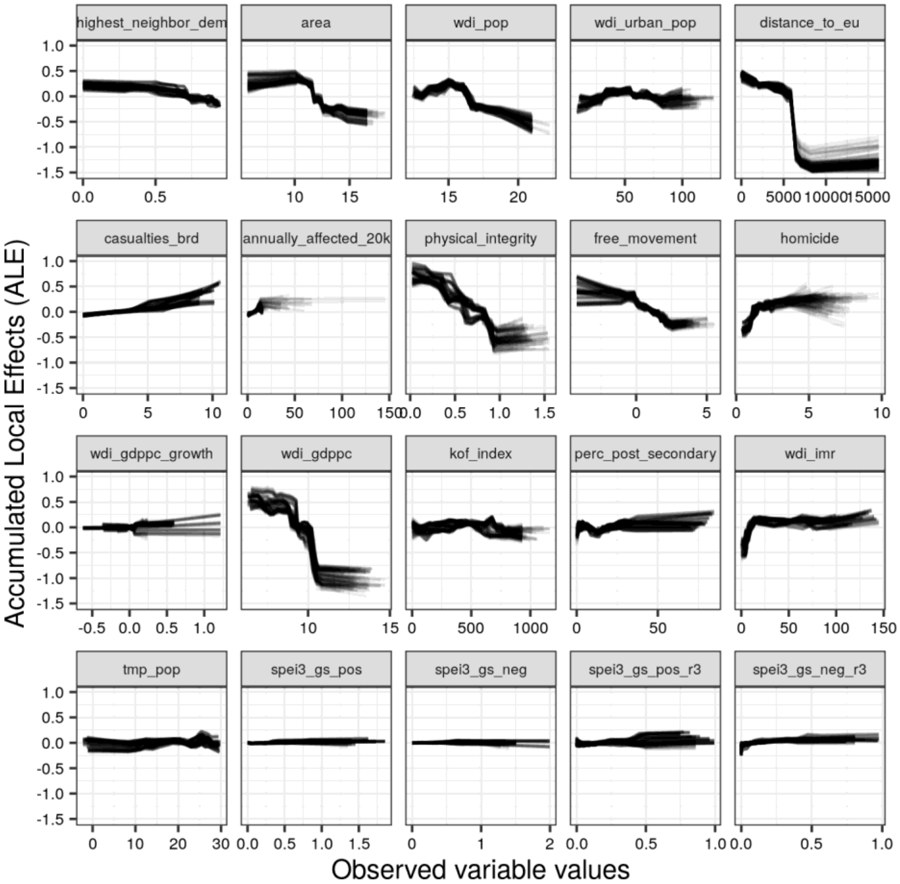

Supplementary Fig. 28. Accumulated Local Effects (ALE); global asylum stocks. ALEs give the marginal difference in prediction with an incremental change in the predictor. Y-axis values represent change in log asylum applications per capita. The results are generated from leave-future-out cross-validation, trained on alternative four-year subsets of empirical data for the period 1999–2017 and tested against observed outcomes for the subsequent year, 2003–2018. N = 3,413 country-year observations, examined over 160 simulations.

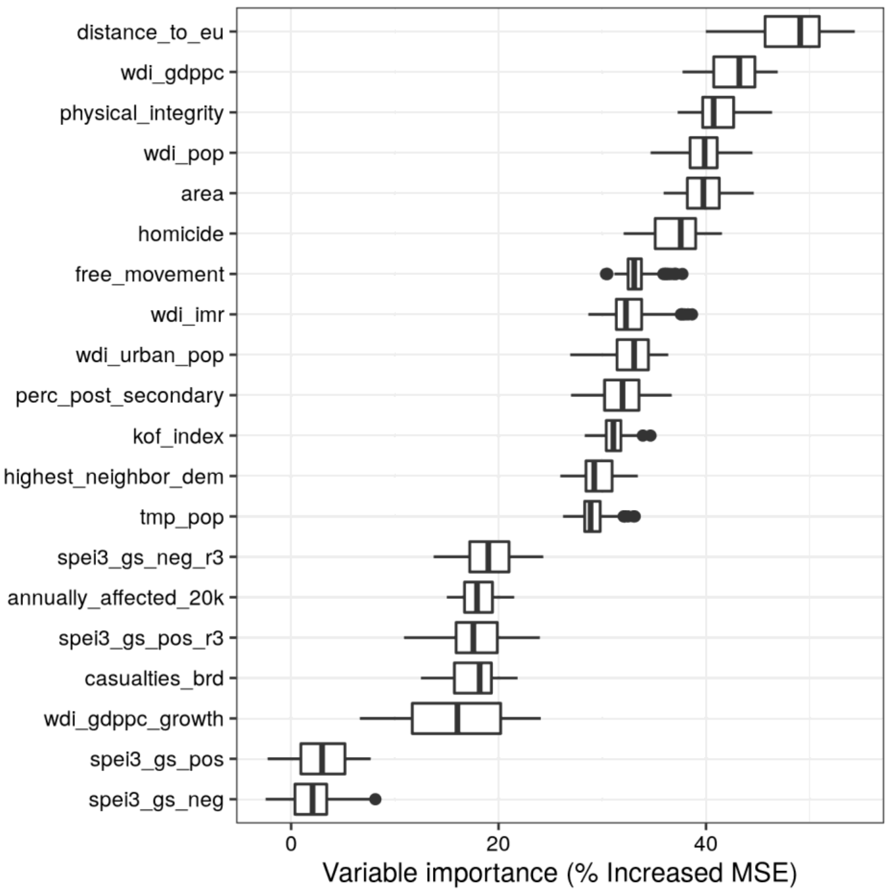

Supplementary Fig. 29. Variable importance plot; global asylum stocks. Boxes range from first to third quartile with interior line marking the median, whiskers denote 1.5 interquartile range, and dots represent outliers. MSE is mean square error. N = 3,413 country-year observations, examined over 160 simulations.

3.10 In-sample regression analysis

In contrast to the predictive approach of this study, much of the relevant empirical literature relies on in-sample regression analysis, typically specifying reduced-form models where all cross-sectional and temporal heterogeneity except for the variable of interest is removed via unit and time constants[2,8–10].

To facilitate comparison with earlier research, we here document the performance of the component indicators in a similar country- and year-fixed effects estimation. However, since the purpose of this design – to estimate causal effects – is qualitatively different from the challenge of identifying factors that predict well on new data, this analysis should not be seen as robustness test of the main results. In the models below, the selection of variables is identical to those comprising the component models in the main specification with exception for the climate component, which now contains an additional squared temperature term (this is not necessary in the LFO-CV models since the random forests explore possible non-linear functional forms for all indicators by design). The inclusive set of indicators introduces considerable multicollinearity, implying that estimated standard errors and p-values may be inflated (again, this is not a concern in the prediction models).

In terms of goodness of fit, the models in Supplementary Table 2 perform similarly – in large part due to the inclusion of common country and year constants that absorb much of the variation in the asylum statistics. Judging by statistical significance (often a poor metric to assess a factor's real effect[11]), we find that virtually all component indicators exert some influence on the dependent variable, and the effects are mostly in line with expectations. The number of people seeking asylum increases as a function of, inter alia: conflict severity, repression, crime, low level of development, and poor economic growth. We also find a U-shaped effect of temperature, similar to that reported by Missirian and Schlenker[2]. Further, we detect a negative migration response to consecutive years of excess wet and dry conditions, possibly reflecting reduced capacity to migrate, as well as a positive association between average education levels and asylum migration to Europe, all else equal. However, as demonstrated in the main analysis, the climate indicators remain comparatively weak when seeking to predict arrivals of asylum seekers on new data.

Supplementary Table. 2. Asylum applications to the EU, 1999–2018

	Violence	Economy	Climate	All
casualties_brd	0.04***			0.03**
	(0.01)			(0.01)
annually_affected_20k	0.01*			0.01
	(0.01)			(0.00)
physical_integrity	-0.71***			-0.63**
	(0.19)			(0.19)
free_movement	-0.14**			-0.14***
	(0.04)			(0.04)
homicide	0.70***			0.56***
	(0.06)			(0.06)
wdi_gdppc_growth		-0.61*		-0.31
		(0.27)		(0.26)
wdi_gdppc		-0.57***		-0.45***
		(0.08)		(0.08)
perc_post_secondary		0.06***		0.05***
		(0.01)		(0.01)
kof_index		-0.00*		-0.00*
		(0.00)		(0.00)
wdi_imr		0.01**		0.00*
		(0.00)		(0.00)
tmp_pop			-0.40***	-0.37***
			(0.08)	(0.08)
tmp_pop_sq			0.01***	0.01***
			(0.00)	(0.00)
spei3_gs_pos			0.02	0.03
			(0.07)	(0.07)
spei3_gs_neg			-0.01	-0.01
			(0.08)	(0.07)
spei3_gs_pos_r3			-0.37**	-0.35**
			(0.13)	(0.12)
spei3_gs_neg_r3			-0.32*	-0.31*
			(0.13)	(0.13)
Intercept	-0.51	5.96*	6.06**	7.67**
	(1.99)	(2.37)	(2.21)	(2.45)
R^2	0.89	0.89	0.89	0.90
Adjusted R^2	0.89	0.88	0.88	0.89
RMSE	0.75	0.76	0.77	0.73
Observations	3,413	3,413	3,413	3,413

Note: Ordinary Least Squares regression coefficients with standard errors in parentheses. Baseline indicators and country and year fixed effects included in all models. $^{***}p < 0.001$, $^{**}p < 0.01$, $^{*}p < 0.05$; two-tailed tests.

References

1. Abel, G. J., Brottrager, M., Crespo Cuaresma, J. & Muttarak, R. Climate, conflict and forced migration. *Glob. Environ. Change* **54**, 239–249 (2019).

2. Missirian, A. & Schlenker, W. Asylum applications respond to temperature fluctuations. *Science* **358**, 1610 (2017).

3. Frelick, B., Kysel, I. M. & Podkul, J. The Impact of Externalization of Migration Controls on the Rights of Asylum Seekers and Other Migrants. *J. Migr. Hum. Secur.* **4**, 190–220 (2016).

4. Crawley, H., Duvell, F., Jones, K., McMahon, S. & Sigona, N. *Unravelling Europe's 'migration crisis' - Journeys over land and sea*. (Policy Press, 2018).

5. Stavropoulou, M. Refugee protection in Europe: time for a major overhaul? *Forced Migr. Rev.* 7–9 (2016).

6. Milner, J. *Refugees, the state and the politics of asylum in Africa*. (Palgrave Macmillan ; in association with St. Antony's College, 2009).

7. UNHCR. *Global Trends: Forced Displacement in 2018*. https://www.unhcr.org/statistics/unhcrstats/5d08d7ee7/unhcr-global-trends-2018.html (2019).

8. Cattaneo, C. & Peri, G. The migration response to increasing temperatures. *J. Dev. Econ.* **122**, 127–146 (2016).

9. Bohra-Mishra, P., Oppenheimer, M. & Hsiang, S. M. Nonlinear permanent migration response to climatic variations but minimal response to disasters. *Proc. Natl. Acad. Sci.* **111**, 9780–9785 (2014).

10. Cai, R., Feng, S., Oppenheimer, M. & Pytlikova, M. Climate variability and international migration: The importance of the agricultural linkage. *J. Environ. Econ. Manag.* **79**, 135–151 (2016).

11. Ward, M. D., Greenhill, B. D. & Bakke, K. M. The perils of policy by p-value: Predicting civil conflicts. *J. Peace Res.* **47**, 363–375 (2010).
