# Supplementary material for: Climatic conditions are weak predictors of asylum migration
Source: Nat Commun. 2021 Apr 6;12:2067. doi: 10.1038/s41467-021-22255-4 (PMC8024373; doi:10.1038/s41467-021-22255-4)
Supplement: Supplementary file 3 — Reporting Summary [file 41467_2021_22255_MOESM3_ESM.pdf]

nature research

Corresponding author(s): Halvard Buhaug

Last updated by author(s): Mar 4, 2021

Reporting Summary

Nature Research wishes to improve the reproducibility of the work that we publish. This form provides structure for consistency and transparency in reporting. For further information on Nature Research policies, see our Editorial Policies and the Editorial Policy Checklist.

Statistics

For all statistical analyses, confirm that the following items are present in the figure legend, table legend, main text, or Methods section.

n/a	Confirmed	
☐	☒	The exact sample size (*n*) for each experimental group/condition, given as a discrete number and unit of measurement
☐	☒	A statement on whether measurements were taken from distinct samples or whether the same sample was measured repeatedly
☐	☒	The statistical test(s) used AND whether they are one- or two-sided<br>*Only common tests should be described solely by name; describe more complex techniques in the Methods section.*
☐	☒	A description of all covariates tested
☐	☒	A description of any assumptions or corrections, such as tests of normality and adjustment for multiple comparisons
☐	☒	A full description of the statistical parameters including central tendency (e.g. means) or other basic estimates (e.g. regression coefficient) AND variation (e.g. standard deviation) or associated estimates of uncertainty (e.g. confidence intervals)
☒	☐	For null hypothesis testing, the test statistic (e.g. F, t, r) with confidence intervals, effect sizes, degrees of freedom and P value noted<br>*Give P values as exact values whenever suitable.*
☒	☐	For Bayesian analysis, information on the choice of priors and Markov chain Monte Carlo settings
☒	☐	For hierarchical and complex designs, identification of the appropriate level for tests and full reporting of outcomes
☐	☒	Estimates of effect sizes (e.g. Cohen's *d*, Pearson's *r*), indicating how they were calculated

Our web collection on statistics for biologists contains articles on many of the points above.

Software and code

Policy information about availability of computer code

Data collection	The dataset was compiled using the statistical software package R, combining indicators from a range of publicly available datasets. The Data Availability section in the article provides details on these sources, including URLs.
Data analysis	Custom open-source R code was developed to conduct the analysis. A link to this code is provided in the Code Availability section in the article.

For manuscripts utilizing custom algorithms or software that are central to the research but not yet described in published literature, software must be made available to editors and reviewers. We strongly encourage code deposition in a community repository (e.g. GitHub). See the Nature Research guidelines for submitting code & software for further information.

Data

Policy information about availability of data

All manuscripts must include a data availability statement. This statement should provide the following information, where applicable:

- Accession codes, unique identifiers, or web links for publicly available datasets
- A list of figures that have associated raw data
- A description of any restrictions on data availability

All underlying data are freely available from the cited sources. The CShapes GIS data of country boundaries are available from https://cran.r-project.org/web/packages/cshapes/index.html. UNHCR statistics on asylum applications by country of origin are available from https://www.unhcr.org/refugee-statistics/download/?url=E1ZxP4. SEDAC's Gridded Population of the World v.4 population raster data are available from https://sedac.ciesin.columbia.edu/data/collection/gpw-v4. Gridded monthly climate data (v.4) from the University of East Anglia's Climate Research Unit are available from https://crudata.uea.ac.uk/cru/data/hrg/. MIRCA 2000 spatial data on crop areas and growing seasons are available from https://www.uni-frankfurt.de/45218023/MIRCA. Country-year statistics on gross domestic product per capita, infant mortality rate, population size, land area, and urbanization are available from the World Bank's World Development Indicators: https://

databank.worldbank.org/source/world-development-indicators. Data on interpersonal globalization are available from ETH Zurich's KOF Globalization Index: https://kof.ethz.ch/en/forecasts-and-indicators/indicators/kof-globalisation-index.html. The Wittgenstein Centre for Demography and Global Human Capital provides country-year data on education levels: http://dataexplorer.wittgensteincentre.org/wcde-v2/. Data on armed conflict (v.19.1) are available from the Uppsala Conflict Data Program: https://ucdp.uu.se/downloads/. Country-year data on individual liberties and electoral democracy (v.9) are available from the V-Dem Institute: https://www.v-dem.net/en/data/archive/previous-data/data-version-9/. The Global Burden of Disease Study 2017 provides statistics on homicide rates: http://ghdx.healthdata.org/gbd-2017. Data on interstate contiguity (v.3.2) are available from the Correlates of War Direct Contiguity Dataset: https://correlatesofwar.org/data-sets/direct-contiguity. Replication data to reproduce results reported here, which were created from these sources, are available from Harvard Dataverse: https://doi.org/10.7910/DVN/6WRMCO.

Field-specific reporting

Please select the one below that is the best fit for your research. If you are not sure, read the appropriate sections before making your selection.

☐ Life sciences ☒ Behavioural & social sciences ☐ Ecological, evolutionary & environmental sciences

For a reference copy of the document with all sections, see nature.com/documents/nr-reporting-summary-flat.pdf

Behavioural & social sciences study design

All studies must disclose on these points even when the disclosure is negative.

Study description	Quantitative simulation of country-level predictors of asylum migration to the EU via leave-future-out cross-validation (LFO-CV) with random forests machine-learning algorithm.
Research sample	The research sample consists of annual observations of independent states, 1999-2018, based on publicly available statistics on countries' economic, political, and climatic conditions. See the Data Availability section for sources of the underlying data.
Sampling strategy	Sampling strategy implied obtaining a complete global sample of independent countries with annual data for all years in the sample period (1999-2018). Simulations were trained on moving four-year subsamples and tested on observations for the subsequent year to assess temporal sensitivity of results while ensuring that all country-years inform calculation of aggregate prediction performance.
Data collection	Data on country-level climatic conditions, economic performance and political characteristics were downloaded from publicly available data repositories at the providing institutions (see Data Availability section for details) and compiled in R. The Author Contribution statement declares which authors were responsible for this task. No blinding was necessary since all data used are derived from widely utilized and reputable providers of country statistics.
Timing	The dataset was compiled on January 6, 2020, drawing on country-level statistics of climatic, economic, and political conditions available at that time. The Data Availability section provides information on specific versions of the underlying datasets.
Data exclusions	No collected data was excluded from analysis.
Non-participation	No non-participation.
Randomization	No randomization was performed. Instead, the LFO-CV procedure draws overlapping four-year subsets of observational data to train the prediction model, which is then tested against observed outcomes for the subsequent year of data. This procedure was repeated for all possible subsets (N=160 simulations in main specification) to ensure that all data points inform the prediction result.

Reporting for specific materials, systems and methods

We require information from authors about some types of materials, experimental systems and methods used in many studies. Here, indicate whether each material, system or method listed is relevant to your study. If you are not sure if a list item applies to your research, read the appropriate section before selecting a response.

Materials & experimental systems

n/a	Involved in the study	
☒	☐	Antibodies
☒	☐	Eukaryotic cell lines
☒	☐	Palaeontology and archaeology
☒	☐	Animals and other organisms
☒	☐	Human research participants
☒	☐	Clinical data
☒	☐	Dual use research of concern

Methods

n/a	Involved in the study	
☒	☐	ChIP-seq
☒	☐	Flow cytometry
☒	☐	MRI-based neuroimaging
